# Non-muscle tropomyosins inhibit myosin-19 and dynamically localize to mitochondrially associated actin filaments

**DOI:** 10.1016/j.jbc.2026.111377

**Published:** 2026-03-18

**Authors:** Cameron P. Thompson, Luther W. Pollard, Mengqi Xu, Erika L.F. Holzbaur, E. Michael Ostap

**Affiliations:** 1 Department of Physiology and Pennsylvania Muscle Institute, Perelman School of Medicine, University of Pennsylvania, Philadelphia, Pennsylvania, USA; 2 Biochemistry Department, University of Nebraska – Lincoln, Lincoln, Nebraska, USA

**Keywords:** myosin, tropomyosin, Actin, actin wave, mitochondria

## Abstract

Myosin-19 (Myo19) is a mitochondrially localized actin-based motor important for regulating mitochondrial homeostasis, including the stabilization of mitochondrial-endoplasmic reticulum (mitoER) contact sites. Thus, proper regulation of Myo19 is likely required to maintain mitochondrial health and function, but little is known about regulatory mechanisms. Non-muscle tropomyosins are known to differentially regulate members of the myosin superfamily, leading us to hypothesize that tropomyosins may regulate Myo19-actin filament interactions. Here, we show that the interaction of Myo19 with actin filaments is inhibited by the association of Tropomyosin 3.1 (Tpm3.1) and 1.7 (Tpm1.7) with F-actin. This inhibition is highly cooperative, as both Tpm isoforms induce an all-or-none stalling of Myo19-driven filaments in *in vitro* gliding assays. In HeLa cells, Tpm3.1 is associated with actin filaments in close proximity to mitochondria. This localization is dynamic, as Tpm3.1 interacts with the mitochondrially-associated actin wave in interphase cells. These findings point toward a tropomyosin-based regulatory mechanism that spatially regulates Myo19 activity in a dynamic manner.

Mitochondria are critical organelles that fuel the cell’s metabolic needs but are also key hubs for cellular signaling pathways (1, 2). Mitochondria respond dynamically to cellular and environmental signals – undergoing tubulation, fission, and fusion. Mitochondria also interact with the cellular cytoskeleton, with long-range motility driven by dynein/dynactin and kinesin motors along microtubules, while shorter range movements and/or local tethering are mediated by myosin motors interacting with actin filaments (AFs) (3, 4, 5, 6).

One such mediator of mitochondrial motility and/or tethering is Myosin-19 (Myo19), a ubiquitously expressed high duty-ratio non-muscle myosin motor (7, 8, 9) that localizes specifically to the outer mitochondrial membrane (OMM) and is a key player in mediating mitochondrial fusion and fission, cristae organization, and mitochondrial motility (5, 10, 11, 12, 13). Myo19 also promotes interorganelle interactions by stabilizing mitoER contact sites (5, 14). Myo19 is recruited to the OMM and remains stably bound to the mitochondria *via* its C-terminal monotopic insertion domain (7, 12, 15). Interactions with Miro1 (Rhot1) and Miro2 (Rhot2) and metaxins 1 and 3 (MTX1, MTX3) may further stabilize the organelle-specific localization of Myo19 (5), while Myo19 motors not embedded in the OMM are rapidly degraded by proteasomes (16). Super-resolution optical imaging suggests that Myo19 is predominantly monomeric when bound to mitochondria, although multiple Myo19 motors can organize together to promote long-range transport along the actin filament bundles in filopodia (5, 9, 11, 17). Mechanisms that regulate Myo19 activity, either actin-based transport or localized organelle tethering, remain to be determined.

Tropomyosins (Tpms) represent a potential regulatory element that could provide spatiotemporal control of Myo19 function in the cell. Tropomyosins are a superfamily of dimeric coiled-coil actin binding proteins that polymerize in a head-to-tail manner along actin filaments (18). Tpms are the products of four genes in humans that can be alternatively spliced to generate 40 or more isoforms (19, 20). These isoforms are generally classified as either short or long, contacting six or seven actin subunits, respectively (21). As a result of an extended binding interface, long isoforms typically bind to actin filaments with higher affinities. Tpms preferentially bind unbranched actin filaments, conferring stability by protecting these filaments from depolymerization by ADF/cofilin and gelsolin (22, 23, 24). Initially described as a component of the sarcomere in muscle (25), non-muscle tropomyosin isoforms are known to play non-redundant functional roles in actin structures such as stress fibers and adhesion complexes, and also display specific localization to organelles like the Golgi (26, 27, 28, 29).

Tpm isoforms have the potential to provide localized, and motor-specific, regulation of myosin activity within the cell. Previously, we have shown that the non-muscle Myosin-1C (Myo1C) undergoes differential modes of regulation by Tpm1.7 (long isoform) and Tpm3.1 (short isoform) (30). While both isoforms inhibit Myo1C-dependent gliding of actin filaments *in vitro*, Myo1C can dislodge Tpm3.1 from actin filaments, while Tpm1.7 remains strongly attached to actin under similar conditions (30). However, this pattern of motor inhibition by Tpms does not always hold; for example, non-muscle Myosin-II is activated by the short isoform Tpm1.8, leading to an increase in the overall ATPase activity of the motor (31, 32, 33). Thus, we wondered whether one or more Tpms might locally regulate Myo19 activity in the context of interactions with mitochondrially-associated actin filaments. Myo19 tethers mitochondria to actin cables in dividing cells, promoting symmetric mitochondrial inheritance (4, 34). These dynamics, termed the actin wave, promote the local fission of mitochondria; subsequent re-fusion in a heterologous manner leads to generalized mitochondrial content mixing, thought to maintain mitochondrial health across the cell population (4, 5, 35). We hypothesized that Tpms might locally regulate the interaction of Myo19 with actin filaments during actin cycling in a spatially specific manner.

Here, we investigate the hypothesis that Tpms regulate Myo19 activity. Structural comparisons predict that Tpm binding will effectively block the interaction of Myo19 with actin filaments. *In vitro* gliding assays confirm that Myo19 is inhibited by Tpm3.1 in a concentration-dependent manner. This inhibition is all-or-none, with a clearly defined boundary between filament gliding and loss of binding to the surface. *In vitro* Tpm3.1 displacement assays demonstrate that Myo19 excludes Tpm3.1 from binding to actin filaments. Cellular experiments show that endogenous Tpm3.1 colocalizes with actin filaments enriched on mitochondria, while exogenously expressed tagged-Tpm3.1 localizes to a circulating actin wave. Taken together, these findings suggest a mechanism by which Myo19 activity is spatially and temporally regulated within the cell by Tpm3.1.

## Results

### Sequence and structural alignments of myosin loop 4s

**Figure 1:**
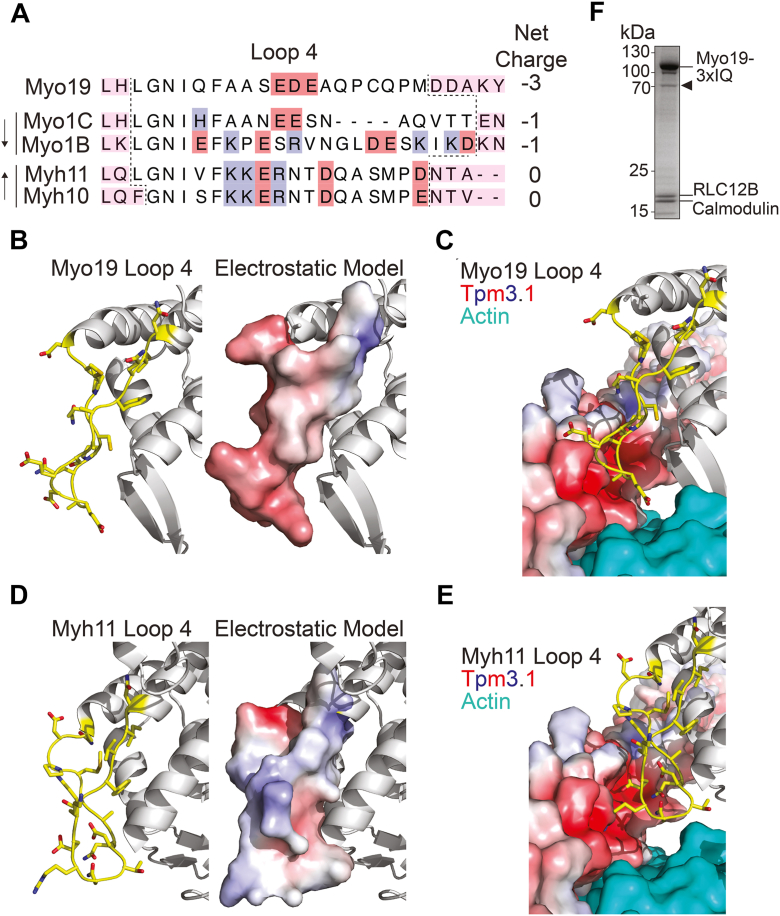
**Sequence and structural alignment of myosin Loop 4 region and expression of Myo19-3xIQ**. *A*, primary structure alignment of Loop 4 region (boundary marked with dashed lines) of Myo19, Myo1C, Myo1B, Myh11 (smooth muscle myosin), and Myh10 (non-muscle myosin II B), motor domains. Charge state of residues highlighted in blue (positive) and red (negative). Magenta boxes represent predicted HL and HM helices that come before and after the Loop 4 region (61). The column of numbers is the net charge of Loop 4 for aligned myosin sequences. Arrows to the left of the chart indicate whether the myosin is inhibited or activated by the presence of Tpms. *B*, predicted structure of Myo19’s Loop 4 as well as its modeled electrostatic surface. *C*, aligned representation of predicted Myo19 Loop 4 if bound to actin (*cyan*) and Tpm3.1 (electrostatic surface). *D*, reported structure of rigor Myh11’s Loop 4 (PDB: 6BIH (40)) as well as its modeled electrostatic surface. E. Aligned representation of reported Myh11 Loop 4 if bound to actin (*cyan*), and Tpm3.1 (electrostatic surface). *F*, Coomassie-stained SDS-Page gel of final elution from FLAG affinity purification protocol containing the Myo19-3xIQ, coexpressed RLC12B and calmodulin light chains, and contaminating EIF-4B (*arrowhead*) protein that contains multiple FLAG-like repeats.

To assess a possible role for tropomyosins in the regulation of Myo19 activity, we performed sequence and structural alignments to compare Myo19’s Loop 4 domain to that of other myosins. Loop 4 has been identified as the critical part of the myosin motor domain that interacts directly with Tpm (36). Alignment of Loop 4 sequences from Myo19, Myo1C, and Myo1B indicate similar net negative charges (Fig. 1*A*). Of note, both Myo1C and Myo1B are inhibited by Tpms (30, 37). In contrast Myh10 and Myh11 are myosin motors that can productively interact with Tpm-decorated actin filaments (31, 38). The Loop 4 sequences of these myosins have a net neutral charge. To determine how the Loop 4 regions of myosins are structurally oriented when the motor is bound to actin, we aligned the actin-bound rigor structures of Myo1C (39) and Myh11 (40) to a recently published structure of a rigor β-cardiac myosin bound to actin and Tpm1.1 (41). All of these structures were aligned using the actin monomers to which the rigor motor was bound given that myosin position on actin filaments varies among the paralogs. We then used AlphaFold3 to predict the structure of Tpm3.1 and aligned this homodimer to the Tpm1.1 molecule in the structure.

The structure of actin-bound Myo19 has not yet been solved, so we used AlphaFold3 to predict its full-length structure but focused on the motor domain and aligned its upper 50 kDa region to actin-bound Myo1B (42). Electrostatic surface modeling revealed that Myo19’s Loop 4 has a net negative charge (Fig. 1*B*) compared to Myh11, which has positively charged patches on its Loop 4 (Fig. 1*C*). Using our alignment strategy, we visualized how the Loop 4 of Myo19 and Myh11 may be oriented when bound to actin, which is interacing with Tpm3.1. Modeling the electrostatic surface of the Tpm3.1 revealed that the negatively charged patches of Myo19 may clash with negatively charged patches on Tpm (Fig. 1*D*) but align with positively charged patches on Myh11 (Fig. 1*E*). Myo1C’s Loop 4, which has similar length and charge distribution as Myo19, is similarly oriented toward a negatively charged patch of Tpm3.1’s electrostatic surface (Fig. S1, *A* and *B*). Myo1B’s Loop 4 is much larger and has a more complex electrostatic surface, leading to both steric and electrostatic clashes with Tpm3.1 (Fig. S1, *C* and *D*). Thus, the charge distribution of Myo19’s Loop 4 may inhibit Myo19 binding to Tpm-actin *via* electrostatic repulsion.

To test this prediction, we expressed a truncated Myo19 lacking the carboxy-terminal tail domain (hereinafter referred to as Myo19-3xIQ; spanning amino acid residues 1–834) (Fig. S2*A*) in SF9 cells along with associated regulatory light chains calmodulin and RLC12B (17, 43). The motor was purified from cell extracts using affinity chromatography (Fig. 1*F*). Motor activity was validated using actin gliding assays. Purified recombinant Myo19-3xIQ drove the gliding of rhodamine phalloidin-labeled actin filaments *in vitro* (Video S1) with an average velocity of 780 ± 71 nm/s. This speed is higher than the previously reported rates of 360 ± 40 nm/s (17) and 229 ± 4 nm/s (8), which may be due to different solution conditions. Previous work has shown that Myo19’s IQ motifs bind calmodulin with different affinities (17). The excess calmodulin in our reaction likely saturated all IQ motifs, stabilizing the lever arm, maximizing the working stroke, and resulting in a faster gliding speed.

### Tpm3.1 inhibits Myo19 activity *in vitro*

**Figure 2:**
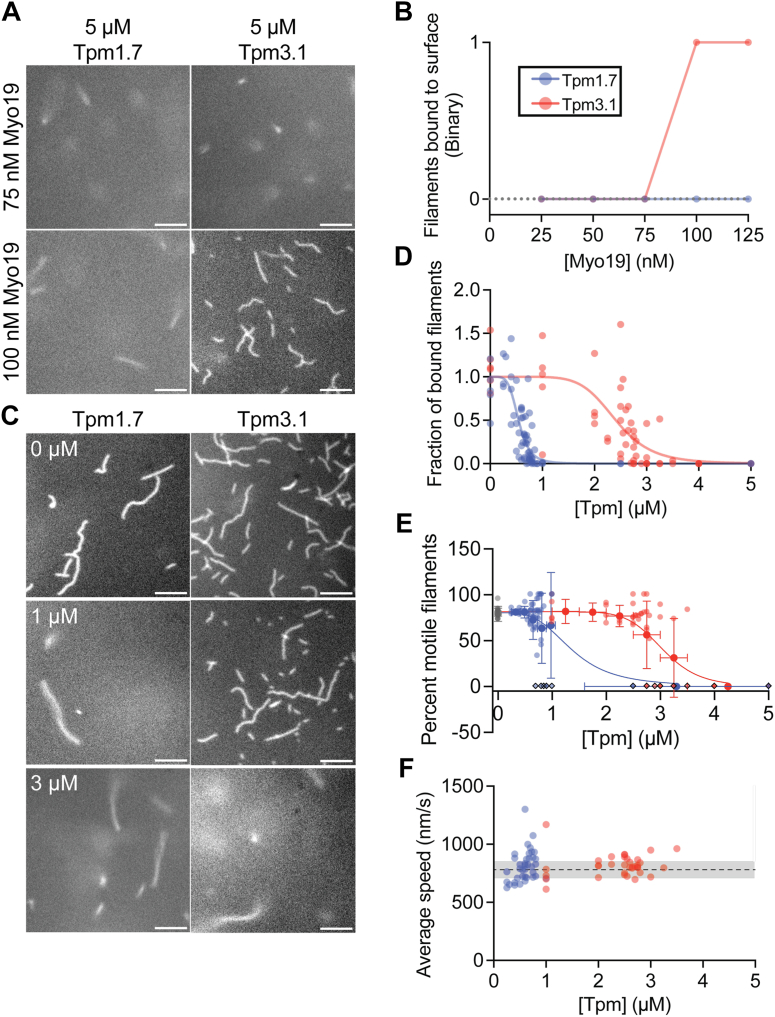
**Both Tpm1.7 and Tpm3.1 show concentration dependent inhibition of Myo19-driven motility**. *A*, representative images of surface-bound actin filaments at saturating concentrations of Tpm1.7/3.1 and 75 nM or 100 nM of Myo19-3xIQ. *B*, binary assessment of presence of surface bound filaments across a range of Myo19 concentrations. *C*, representative images of actin filaments bound to a Myo19-3xIQ coated surface at either 0 μM, 1 μM, or 3 μM of Tpm1.7/3.1. *D*, fraction of surface-bound filaments normalized to 0 μM Tpm1.7/3.1 control as a function of Tpm concentration. *E*, percentage of motile filaments normalized to 0 μM Tpm1.7/3.1 controls as a function of Tpm concentration showing all data points (shaded) and averages of binned data (solid points), diamond outlined points indicate no filaments were bound to the surface. *F*, average speed of motile surface-bound filaments across a range of Tpm1.7/3.1 concentrations, solid line represents the average speed of 0 μM Tpm control chambers and shaded region indicate ± 1 standard deviation.

Based on our structural predictions that Tpm could influence Myo19’s interaction with actin filaments, we tested whether Tpm impairs Myo19 motor activity using *in vitro* actin gliding assays. Given the similarity between Myo19’s Loop 4 and that of Myo1C, we replicated the *in vitro* Tpm-regulation experiments previously performed with Myo1C (30). We examined two isoforms: Tpm3.1, a short isoform abundantly expressed in cancer cells including HeLa (44), and Tpm1.7, a representative long isoform (Fig. S2*E*). Increasing concentrations of purified Myo19-3xIQ were bound to the surface of a flow chamber, and following several washes, we added a final solution containing labeled F-actin and recombinantly expressed Tpm1.7 or Tpm3.1 (Fig. S2, *B* and *C*) at concentrations to ensure actin was Tpm-decorated (30). Robust actin gliding was observed in the presence of 25 to 125 nM Myo19 in time-lapse videos acquired at 37 °C (Video S2. In contrast, no actin gliding was observed in the presence of either Tpm1.7 or Tpm3.1 at Myo19-3xIQ concentrations less than 75 nM, suggesting that Tpm1.7 and Tpm3.1 each inhibit the interaction of Myo19 with F-actin under these conditions. Intriguingly, at higher concentrations (>75–100 nM) of Myo19-3xIQ, Tpm3.1 inhibition was overcome, and actin gliding was observed (Fig. 2*A*). A titration curve showing the all-or-nothing binding of actin filaments to the surface suggests a Myo19 concentration-dependent regulation by Tpm3.1 but not by Tpm1.7 in the range of Myo19 concentrations we tested (Fig. 2*B*).

Next, we examined how the Tpm concentration affects Myo19-3xIQ-driven actin gliding. Using Myo19-3xIQ surface-immobilized *via* neutravidin, we introduced labeled F-actin with varying concentrations of Tpm1.7 or Tpm3.1. Increasing Tpm concentrations caused a shift from robust, smooth gliding to complete inhibition of filament binding, with IC_50_ value of 0.54 μM with a 95% confidence interval of 0.49 μM – 0.59 μM for Tpm1.7 and 2.41 μM with a 95% confidence interval of 2.05 μM – 2.6 μM for Tpm3.1 (Fig. 2, *C* and *D*). In these assays, we observed a very sharp boundary between bound, gliding filaments and no filaments bound at all (Fig. 2*E*), suggesting that the inhibition is behaving in an all-or-none manner. Indeed, we found that all moving filaments travel at a speed similar to sliding velocities observed in the absence of tropomyosin (Fig. 2*F*). In contrast, Tpm3.1 inhibits Myo1C through a reduction in gliding speed without disrupting filament binding; actin filaments remain attached and motile even at high Tpm3.1 concentrations, as we reported previously (30). Collectively, these results demonstrate that Myo19-driven actin gliding is inhibited by the presence of Tpm1.7 or Tpm3.1 in a concentration-dependent manner, leading us to hypothesize that Myo19 and Tpm3.1 directly compete for binding on actin.

### Myo19 and Tpm3.1 compete for binding on actin

**Figure 3:**
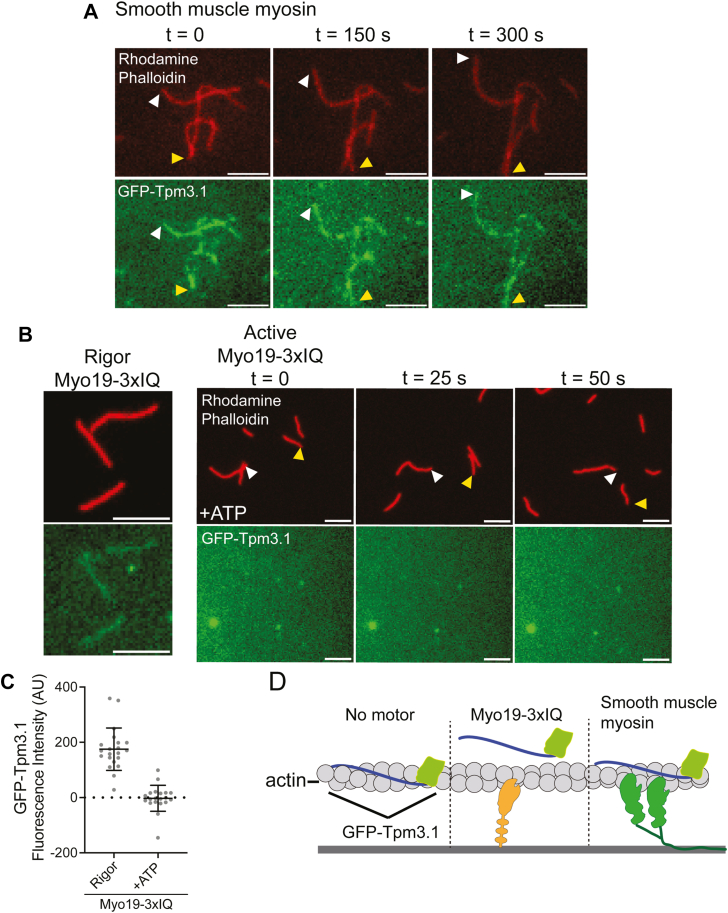
**Myo19-Tpm3.1 and Tpms compete for actin binding**. *A*, Time-lapse images depicting smooth muscle myosin–driven motility of rhodamine–phalloidin–stabilized actin filaments decorated with GFP–Tpm3.1 (5 μm scale bar). *B*, *left*. Still frame of rigor Myo19-3xIQ coated surfaces in the presence of GFP-Tpm3.1 coated rhodamine phalloidin stabilized actin filaments (5 μm scale bar). *B*, *right*. Series of time-lapse images showing Myo19-3xIQ driven gliding of rhodamine phalloidin stabilized actin filaments in the presence of ATP and the lack of corresponding GFP-Tpm3.1 signal (5 μm scale bar). *C*, quantification of GFP-Tpm3.1 signal that overlaps with rhodamine phalloidin signal for Myo19-3xIQ with or without ATP (Mean ± 1 Stdev.). *D*, graphical representation of TIRF results where GFP-Tpm3.1 binds to actin in the absence of motor, competes for binding with Myo19-3xIQ, and bind simultaneously with smooth muscle myosin.

Different myosins exhibit distinct interactions with Tpm-decorated actin filaments; some can bind the Tpm–actin complex, while others compete with Tpm for access to F-actin. For example, smooth muscle myosin binds Tpm1.4-decorated filaments and increases the rate of actin gliding (45), whereas Myo1C displaces Tpm3.1 upon binding (30). We tested whether Myo19 also competes with Tpm3.1 for actin binding. We used total internal reflection fluorescence (TIRF) microscopy to visualize the binding of GFP–Tpm3.1 to actin filaments. As a control, smooth muscle myosin S1 was immobilized on a glass coverslip, and pre-assembled GFP–Tpm3.1–rhodamine-phalloidin–actin complexes were introduced in the presence of ATP. Overlapping GFP and rhodamine signals confirmed that GFP–Tpm3.1 colocalizes with gliding actin filaments propelled by smooth muscle myosin (Fig. 3*A*). In the absence of ATP, GFP-Tpm3.1 remained colocalized with rhodamine-labeled actin filaments on rigor Myo19-3xIQ-coated surfaces, indicating that Myo19 can bind to Tpm3.1-decorated actin in the rigor state (Fig. 3*B*). However, this does not exclude the possibility of competition, as the limited number of strong-binding sites in rigor may be insufficient to displace Tpm3.1, and limited dissociation would not be detectable by our assay. To test whether active cycling alters this interaction, we repeated the experiment in the presence of 5 mM MgATP. Under these conditions, we observed robust actin filament gliding, but the GFP signal was absent from gliding filaments, indicating that Tpm3.1 was dislodged as Myo19 engaged actin during its mechanochemical cycle (Fig. 3*B*). Quantification of GFP-Tpm3.1 signal colocalized with actin confirmed a substantial loss of fluorescence upon ATP addition across two independent Myo19-3xIQ preparations (Fig. 3*C*). These findings suggest that Myo19 displaces Tpm3.1 during active cycling, consistent with a model of direct competition for actin binding (Fig. 3*D*).

### Tpm3 localizes with mitochondrially-associated actin filaments

**Figure 4:**
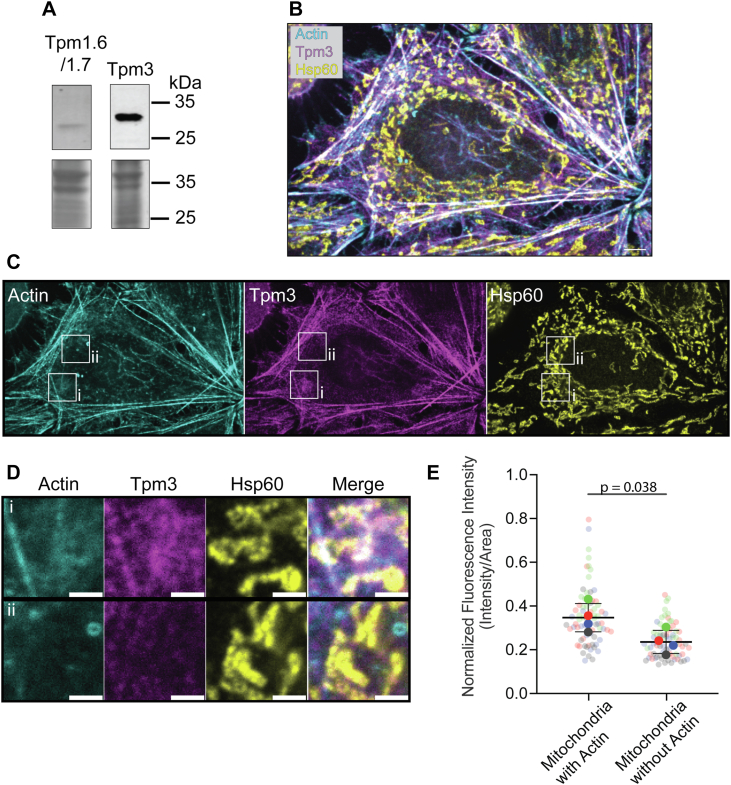
**Endogenous Tpm3 is enriched on mitochondrial-associated actin filaments**. *A*, Western blot analysis (*Top*) of endogenous expression of Tpm1.6/1.7 and Tpm3 in HeLa lysates, dashed line indicates predicted size od Tpm1.6/1.7, bottom images are corresponind total protein stains. *B* and *C*, representative micrograph of fixed HeLa cells with F-actin labeled using rhodamine-phalloidin (*cyan*), and Tpm3 (*magenta*) and mitochondria (*yellow*) labeled using corresponding antibodies (5 μm scale bar). *D*, inset images showing the localization of actin and Tpm3 near mitochondria when the actin wave is (i) or is not (ii) present (2 μm scale bar). *E*, quantification of endogenous Tpm3 fluorescence signal overlapping with mitochondria that are or are not surrounded by the actin wave (Mean ± 1 Stdev.).

Having established that Tpm regulates Myo19 activity *in vitro*, we next asked whether Tpm1.7 and Tpm3.1 localize to actin structures that Myo19 may interact with in cells. Using HeLa lysates, we detected strong expression of Tpm3 (∼30 kDa), which is predominantly Tpm3.1 in HeLa cells (44), and no detectable signal for Tpm1.6/1.7 (∼32.7 kDa) (Fig. 4*A*), consistent with previous reports of Tpm isoform abundance in this cell type (44). Given this finding, we examined the endogenous cellular organization of actin and Tpm3 near mitochondria. HeLa cells were fixed, permeabilized, and incubated with primary antibodies against Tpm3 and Hsp60, a mitochondrial marker (Fig. 4, *B* and *C*). We found that Tpm3 was localized to stress fibers and peripheral actin structures, as previously reported (29). Furthermore, we identified a subpopulation of actin enriched on mitochondria, where Tpm3 also co-localized (Fig. 4*D*). The normalized fluorescence intensity of the Tpm3 overlapping with Hsp60 is higher for mitochondria that are surrounded by actin (Fig. 4*E*). Together, these results suggest that Tpm3 is recruited to mitochondrially associated actin filaments.

### A Tpm3.1 wave circulates the cell with the actin wave

**Figure 5:**
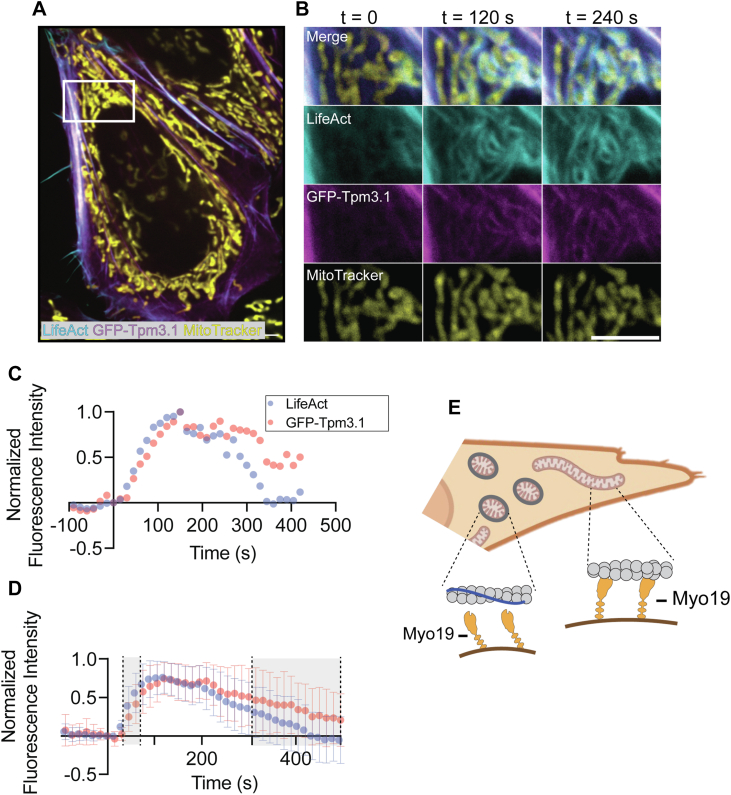
**A Tpm3.1 wave traverses the cell concurrently as the actin wave**. *A* and *B*, representative micrograph images showing full frame merge along with inset merge and individual channel images from long-term timelapse videos of HeLa cells overexpressing LifeAct mScarlett (*cyan*), GFP-Tpm3.1 (*magenta*), and mitochondrial labeled with mitoTracker DeepRed (*yellow*) at an initial time-point and 120 and 240 s later (5 μm scale bar). *C*, individual fluorescence trace of LifeAct-mScarlett (*blue*) and GFP-Tpm3.1 (*red*) as it traverses a region of the cell. *D*, ensemble averages of many individual traces aligned at a point prior to actin-wave fluorescence intensity increase show simultaneous appearance of LifeAct-mScarlet and GFP-Tpm3.1 signal (Mean ± 1 Stdev.). Shaded region denotes timepoints where the Mann–Whitney *U* test determined there was significant difference in the rate of dispersion. *E*, schematic depicting the role of Tpm3.1 in the actin wave determining which population of actin Myo19 can or cannot interact with.

To explore the dynamics of Tpm3.1 and actin filaments in the cell, we pursued live cell-imaging of HeLa cells overexpressing GFP-Tpm3.1 (29) (Fig. S2*D*) and LifeAct-mScarlet (46), which binds to F-actin. Time-lapse videos were captured (4 frames/min for 15 min, Video S3 and Fig. S3) to observe the overall dynamics of the actin architecture in the cell. As described previously (34, 35), a mitochondrially-associated actin wave was observed navigating the cell (Fig. 5, *A* and *B*). In parallel, a GFP-Tpm3.1 wave was observed traversing the cell with the actin wave, enveloping mitochondria as it cycled through the cytoplasm.

To gain quantitative insights into the dynamics of this actin-Tpm wave, the fluorescence intensity of both LifeAct-mScarlet and GFP-Tpm3.1 channel were monitored as the wave visibly traversed a region of the cell (Video S4). Following background correction and normalization (described in Methods, visualized in Fig. S4, *A*–*E*), the intensity traces were aligned at the inflection point immediately preceding the appearance of the wave (Fig. 5*C*). The average normalized fluorescence intensity for each time-point after the inflection was determined across many measured regions of interest. This ensemble average approach was used to gain insight into the relative timing of F-actin assembly/disassembly and Tpm3.1 recruitment and dispersal (Fig. 5*D*). As the wave traversed the measured region, the actin signal peaked before the GFP-Tpm3.1 signal, suggesting that Tpm does not occur concurrently with polymerization. After the signal plateaus, the GFP-Tpm3.1 signal disperses at a slower rate than the actin (Fig. 5*D*, shaded regions; Fig. S4*F*). The difference in rates of appearance or dispersion can provide insights into the underlying architecture of the actin wave. Since Tpms prefentially bind and stabilize linear actin (22, 23, 24), our data suggest that branched actin polymerization proceeds linear actin as the wave initially surrounds a mitochondrion. Conversely, as the wave exits an area the linear actin filaments remain polymerized longer, stabilized by the bound Tpms. Taken together, these findings point toward the localized, transient assembly of Tpm3.1 on mitochondrially-associated actin filaments that negatively regulates Myo19 tethering or motor activity on mitochondria within the actin wave (Fig. 5*E*).

## Discussion

Here we report that Myo19 activity is regulated by tropomyosins, with both Tpm1.7 and Tpm3.1 inhibiting actin gliding *in vitro*, though with distinct behaviors. Structural modeling predicts that Myo19’s Loop 4 architecture leads to inhibition, and our biochemical assays confirm concentration-dependent regulation. Importantly, we find that Myo19 can actively displace Tpm3.1 from actin filaments during its mechanochemical cycle, Tpm3.1 localizes to mitochondria-associated actin filaments and cycles dynamically with the interphase actin wave. Together, these findings suggest that Tpm3.1 is a spatiotemporal regulator of Myo19, providing new insight into how actin–motor interactions are tuned in cellular contexts critical for mitochondrial function.

The conserved, repetitive biochemical properties of Tpm surface residues (21, 47) suggests that the inhibition of Myo19 would be observed for other isoforms not tested in this work. Our sequence and structural alignment analyses suggest a basis for why Myo19 is inhibited by tropomyosins (Fig. 1, *B* and *C*). The Loop 4 region of Myo19, like that of Myo1C, includes a region of negatively charged residues. When oriented on actin, these charges appear to be positioned to clash with negatively charged surfaces of Tpm3.1, likely preventing stable binding. In contrast, myosins such as Myh11, which can function on Tpm-decorated filaments, have positively charged Loop 4 regions oriented away from this surface (40). Prior studies suggest that Loop 4 can form stabilizing contacts with actin (39, 42), which may further constrain the orientation of Myo19’s Loop 4 in a way that enhances electrostatic clashes with Tpm. These features together provide a structural explanation for why Myo19, like Myo1B and Myo1C, is inhibited by tropomyosins, while others, like Myh11, are accommodated.

Our assays show that concentration-dependent inhibition of Myo19 differs between Tpm isoforms. Both long (Tpm1.7) and short (Tpm3.1) isoforms inhibit Myo19-driven gliding, but with 5-fold differences in their IC_50_ values (Fig. 2*D*). The stronger inhibition by Tpm1.7 likely reflects its extended actin-binding footprint, resulting in higher actin affinity. Myo19 is able to overcome Tpm3.1 inhibition at higher motor concentrations, whereas Tpm1.7 inhibition was not overcome under the conditions tested. Strikingly, inhibition of Myo19 occurs in an all-or-none manner. Filaments either glide at normal speed or fail to bind entirely, without the graded slowing observed for Myo1C (30). This cooperative, threshold-like inhibition is reminiscent of the Tpm-dependent regulation of muscle myosin-II (36, 48), suggesting that Myo19 regulation may employ a similar gating mechanism to restrict activity to specific filament populations.

We hypothesize that Myo19 and tropomyosins compete for to actin filaments, and that this competition underlies the all-or-none regulatory behavior we observe. Our TIRF competition assays support this idea, showing that Myo19 can displace Tpm3.1 when in its strong, actin-bound state (Fig. 3, *B* and *C*). The apparent discrepancy between rigor-state and actively cycling Myo19 can be explained by the effective concentration of actin-bound motors. A rigor Myo19 interacts only once with the filament, displacing local Tpm without disrupting the cooperatively bound polymer, whereas an active motor continuously rebinds, raising the effective actin-binding concentration and thereby outcompeting Tpm. This interpretation, together with the Myo19 titration experiments (Fig. 2, *A* and *B*), supports a model in which a threshold concentration of Myo19 is sufficient to overcome Tpm3.1 inhibition.

Tpms localize to distinct populations of actin in the cell (20). We found that Tpm3 localizes to actin filaments that transiently polymerize and depolymerize around mitochondria, facilitating functions like organelle motility, fission, fusion, or tethering. (Fig. 4). Using live-cell imaging, we explored the interphase actin wave and found that GFP-Tpm3.1 cycles with the actin wave (Fig. 5, *A*–*D*). Quantitative analysis of local assembly/disassembly events demonstrates that Tpm3.1 appearance slightly lags the initial assembly of actin around a mitochondrion. However, the actin signal disperses faster than the GFP-Tpm3.1 signal as the wave exited our measured region of interest. Given the knowledge that Tpms preferentially bind to linear actin (22, 23, 24), our findings agree with the previously reported dynamic nature of the actin that comprises the wave (34, 35, 49). Our data suggests that the initial polymerization by Arp2/3 generates a Tpm-free population of branched actin which Myo19 could interact with to drive the wave-induced mitochondrial fission. Subsequently, formins promote the polymerization of linear, Tpm-coated actin filaments that are more stable and longer-lasting. The change in ratio of primarily Tpm-free to Tpm-coated filaments may act as a way to limit Myo19’s activity, preventing unchecked mitochondrial fission which would be deleterious to the cell. Further investigation is required to understand how the cell regulates Tpm inhibition of Myo19 spatiotemporally to selectively enable or disable actin-Myo19 interactions.

## Experimental procedures

### Proteins

Myo19-3xIQ (Fig. 1*A*), described previously (17), includes an N-terminal FLAG (DYDDDDK)-TEV tag, amino acids 1 to 834 from human Myo19 (UniprotID: Q96H55), and a C-terminal BioTag which allows for biotinylation within the cell (50). The motor was expressed in Sf9 cells along with calmodulin (CaM) and RLC12B with an excess 0.2 mg/ml of biotin in growth media. The protein was purified using either a HEPES lysis buffer (25 mM HEPES pH 7.5, 300 mM NaCl, 5 mM MgCl_2_, 1 mM EGTA, 0.5% IGEPAL, 25 mM sucrose, 1 mM PMSF, 2 mM DTT, 2 mM ATP, protease inhibitors) or MOPS lysis buffer (20 mM MOPS pH 7.5, 130 mM KCl, 5 mM MgCl_2_, 2 mM EGTA, 5% glycerol, 0.05% NP-40, 2 mM ATP, 0.5 mM DTT, 2 μM CaM, protease inhibitors (9)). The two different purification buffers were used with the aim of improving protein yield but yield and activity of the resulting protein were the same. Lysate was clarified at 138,000×*g* (based on average radius for Beckman Ti-45 rotor) for 1 h at 4 °C. The supernatant was flowed over column containing FLAG resin (Genscript: L00907–10) for 1 h at 4 °C followed by washes with a HEPES or MOPS wash buffer with and without 2 mM ATP (HEPES: 50 mM HEPES pH 7.5, 300 mM NaCl, 5 mM MgCl_2_, 1 mM EGTA, 2 mM DTT, protease inhibitors, MOPS: 20 mM MOPS pH 7.5, 75 mM KCl, 5 mM MgCl_2_, 2 mM EGTA, 0.5 mM DTT, protease inhibitors). Protein was eluted from the column in three steps after 1 h, 30 min, and 5 min incubation with 0.5 mg/ml FLAG peptide diluted in either HEPES or MOPS elution buffer (HEPES: 50 mM HEPES pH 7.5, 300 mM NaCl, 5 mM MgCl_2_, 1 mM EGTA, 2 mM DTT, 10 μM CaM, protease inhibitors, MOPS: 20 mM MOPS pH 7.5, 75 mM KCl, 5 mM MgCl_2_, 2 mM EGTA, 0.5 mM DTT, 0.5 mM ATP, 10 μM CaM, protease inhibitors). Eluted fractions were pooled and dialyzed *versus* 50% glycerol diluted in HEPES or MOPS solution (HEPES: 50 mM HEPES pH 7.5, 300 mM NaCl, 1 mM EGTA, 2 mM DTT, MOPS: 20 mM MOPS pH 7.5, 75 mM KCl, 5 mM MgCl_2_, 2 mM EGTA, 0.5 mM DTT, 0.5 mM ATP). Final Myo19 concentrations were determined by densitometry of SDS-PAGE gels stained with Coomassie Blue compared to known masses of Bovine Serum Albumin (Sigma-Aldrich: A7906–50G). A ∼70 kDa contaminant was observed in all Myo19-3xIQ preparations. Using Mass Spectrometry, this protein was identified as eukaryotic translation initiation factor 4B (EIF-4B), whose amino acid sequence contains FLAG-like stretches of sequences rich in aspartic acids, likely explaining its enrichment through affinity chromatography. The contaminant was most likely eliminated during the wash step following the immobilization of biotinylated Myo19 on neutravidin-coated motility surfaces. (see below).

Acetyl-mimic Tpm1.7 and Tpm3.1 proteins with an acetyl mimicking N-terminal M-A-S extension, were expressed and purified from *Escherichia coli* following established methods (51). Superfolder (msf)EGFP N-terminally linked with the acetyl mimicking sequnce M-A-S to Tpm3.1 was expressed and purified as described previously (29, 30). Smooth muscle myosin S1 was prepared and used in *in vitro* gliding assays as described previously (45), a Myc antibody (ThermoFisher, 9E10) was used to bind the purified motor to the surface. Rabbit skeletal muscle actin was purified following previously a published protocol (52) and was polymerized and stabilized in the presence of fluorescent (Invitrogen, R415) or unlabeled (Cayman Chemicals, #18039) phalloidin.

### *In vitro* gliding motility

Nitrocellulose-coated flow chambers were constructed as described (17). Briefly, neuturavidin (0.1 mg/ml) was adsorbed to the nitrocellulose-coated surface for 3 min, followed by surface blocking with 2 mg/ml casein for 3 min. After washing 2 times with 100 μl, varied concentrations of biotinylated Myo19 in M19-Buffer (25 mM HEPES pH 7.5, 300 mM NaCl, 5 mM MgCl_2_) were bound to neutravidin. Motility was initiated upon the addition of 10 nM fluorescently-labeled actin filaments in KMg25 (10 mM MOPS pH 7, 25 mMKCl, 1 mM EGTA, 1 mM MgCl_2_) plus 2 mM MgCl_2_ supplemented with components necessary for filaments motility (5 mM ATP, 20 mM DTT, 5 mg/ml glucose, 0.2 mg/ml glucose oxidase, excess (>30 μM) CaM). Acetyl-mimic Tpm1.7 or Tpm3.1 was added to the motility solution to achieve a desired final Tpm concentration. Chambers were allowed to equilibrate to 37 °C for 2 min prior to capture of time-lapse videos (200 ms exposure time, one frame per second) with a duration of 30 s. Fluorescent actin filaments were visualized using a Leica DMIRB microscope with a 100x Leica oil-immersive objective of numerical aperture 1.4. Velocity analysis was performed using the Manual Tracking plugin in ImageJ (53). For Tpm titration experiments, the surface-bound Myo19 concentrations ranged from 275 nM to 600 nM.

For TIRF microscopy motility assays, flow chambers were coated with either Myo19-3xIQ or smooth muscle myosin S1. The chamber surface was then blocked with 10 mg/ml casein, and reaction mixtures containing unlabeled or GFP-Tpm3.1 and 0.2 mg/ml casein were introduced as described above. Images were acquired at room temperature. Fluorescent actin filaments and Tpm molecules were visualized using a Nikon Ti2E microscope with a 60x Apochromat TIRF objective with N.A. 1.49 running NIS-Elements.

GFP-Tpm3.1 fluorescence signal overlap with rhodamine phalloidin was determined by first drawing two line segments in the actin channel, one directly over the filament and another just off the filament. These line segments were used to measure the fluorescence intensity in the GFP channel with the line on the actin representing actin-bound Tpm3.1 and the line just off the actin used to correct for background signal. The actin-bound signal was correcting using the background signal and the resulting value was plotted. 10 filaments were measured across two sets of experiments.

### Cell and imaging

HeLa-M cells (A. Peden, Cambridge Institute for Medical Research) were grown in a 37 °C, 5% CO_2_ incubator in Dulbecco's modified Eagle's medium (Corning; catalog no.: 10–017-CV) supplemented with 10% fetal bovine serum and 1% GlutaMax (Gibco; catalog no.: 35,050,061) and passaged using trypsin. Prior to imaging, cells were plated on #1.5 glass-bottom dishes. Transfections and labeling were performed according to the manufacturer’s instructions. Briefly, plasmids were diluted in OPTI-MEM (Gibco; catalog no. 31985–070) to a volume equivalent to 10% of the maintenance medium and mixed with FuGENE six transfection reagent (Promega; catalog no. E269 A) for 15 min at room temperature. The mixture was added to cells, which were incubated for 24 h to allow expression. Cells were then incubated in maintenance medium containing MitoTracker Deep Red FM (1:4000 dilution) for 10 min, washed once, and transferred to imaging medium consisting of Leibovitz’s medium (Gibco; catalog no. 11415–064) supplemented with 10% fetal bovine serum and 1% GlutaMAX.

For immunocytochemistry of endogenous Tpm3 expression, HeLa-M cells were seeded on #1.5 glass-bottom dishes and fixed in 4% paraformaldehyde and 4% sucrose in PBS for 10 min at 37 °C followed by three washes with PBS. Cells were permeabilized with 0.2% Triton in PBS for 15 min at room temperature. Imaging dishes were washed three more times with PBS and then blocked with Blocking Solution (1% bovine serum albumin (BSA), 5% goat serum in PBS) for at least 90 min at room temperature. Cells were incubated with primary antibodies for proteins of interest diluted in Blocking Solution at 4 °C overnight. Primary antibodies used with 4% paraformaldehyde and 4% sucrose fixation include Tpm3 (anti-Mouse, DSHB – CG3-S, 1:1000 dilution) and Hsp60 (anti-Chicken, Invitrogen – PA5-143571, 1:2000 dilution). Dishes were washed three times with PBS and then protected from light while incubating for an hour at room temperature with species-matched secondary antibodies (1:500 dilution) and 165 nM rhodamine phalloidin in Blocking Solution. Following three PBS washes, coverslips were mounted in Aqua-Poly/Mount (Polysciences – 18,606–20). Images were acquired as single planes or Z-stacks with 200 nm step-size. To quantify endogenous Tpm3 near mitochondria, the mitochondrial and actin channels were used to identify organelle populations within the same cell that either lacked detectable actin or were surrounded by the cloud-like actin morphology characteristic of the actin wave. A small ROI was drawn around each mitochondrial population, and Fiji was used to measure fluorescence intensity in the actin and Tpm3 channels. Signals were normalized to ROI area. Four independent experiments were performed, with 18 to 20 cells analyzed in each experiment.

Confocal microscopy was conducted using an UltraView VoX PerkinElmer spinning-disk system on a Nikon Eclipse Ti Microscope with an 100 × Apochromoat objective with N.A. 1.49 (Nikon) and an EM-charge-coupled device camera (C9100; Hamamatsu Photonics). Microscope acquisition parameters were kept constant, and cells were imaged at random. Time-lapse videos were captured at either four frames per minute for 15 min or one frame per second for 5 min to capture wave trajectory in the cell or actin wave dynamics, respectively.

Videos were analyzed in Fiji (54), and fluorescence intensity was measured for each channel across the entire image or within a defined region of interest through which an active wave passed. Photobleaching was corrected by normalizing whole-image intensity traces to the initial time point and applying the fractional decrease at each frame to adjust intensities within the region of interest. Region of Interest intensity traces were baseline-subtracted so that the value immediately before the actin wave appearance was set to zero, and all subsequent values were normalized to subsequent plateau (Fig. S4). Ensemble averages were generated by aligning all zero time points across videos and calculating the mean and standard deviation. Mann-Whitney analysis was performed to compare the actin and GFP-Tpm3.1 average fluorescence plots (Fig. S4*F*). Cliff’s Delta was calculated manually (55, 56). These two values were plotted for time-points post-inflection up until t = 595 s, after wich there were not enough replicates to perform statistical analysis. The average intensity of actin and GFP-Tpm3.1 were classified as significantly different if the Cliff’s Delta was above a value of 0.3 (denoted by the horizontal black line) and also had a *p*-value < 0.05 (denoted by the red horizontal line).

To quantify endogenous Tpm3 fluorescence around mitochondria within or outside the actin wave, the Hsp60 channel was thresholded using the Otsu method in Fiji. This thresholded mask was then applied to measure Tpm3 fluorescence intensity.

### Immunoblotting endogenous Tpm expression

HeLa-M cells were incubated in RIPA lysis buffer (50 mM Tris-HCl [pH 7.4], 150 mM NaCl, 1% Triton, 0.5% Deoxycholate, 0.1% Sodium dodecyl sulfate (SDS)) for 30 min on ice. Lysates were then spin clarified at 17,000×*g* for 10 min at 4 °C. Lysates were run on a 4%-20% SDS-PAGE gels transferred to Immobilon-FL PVDF membranes (Millipore; 05,317) using a wet blot transfer system (BioRad). Membranes were then stained for total protein using LI-COR Revert 700 Total Protein Stain and imaged using an Odyssey CLx Infrared Imaging System (LI-COR). Following imaging of total protein stain, membranes were de-stained and blocked for 5 min with EveryBlot Blocking Buffer (BIO-RAD; 12010020). Membranes were incubated with primary antibodies for Tpm1.6/1.7 (anti-Mouse, DSHB – Cgbeta6-S, 1:1000 dilution) and Tpm3 (anti-Mouse, DSHB – CG3-S, 1:1000 dilution) in EveryBlot at 4 °C overnight. Membranes were washed three times in TBS (50 mM Tris-HCl [pH 7.4], 274 mM NaCl, 9 mM KCl) with 0.1% Tween-2 (TBST) and incubated with species-matched secondary antibodies diluted 1:20,000 in EveryBlot with 0.01% SDS for 1 h at room temperature. Membranes were washed three times with TBST and images on Odyssey CLx Infrared Imaging System. Western blots were analyzed with Image Studio Software (LI-COR).

### Sequence and structural alignment

The amino acid sequences of Myo19 (Uniprot: Q96H55), Myosin-1C (Uniprot: O00159), Myosin-1B (Uniprot: O43795), Non-muscle myosin IIB (Myh10, Uniprot: P35580), Smooth muscle myosin (Myh11, Uniprot: P35749), and β-cardiac myosin (Myh7, Uniprot: P12883) were aligned using the NCBI’s Constraint based Multiple Alignment Tool (COBALT) online tool (57). The resulting alignment was analyzed using Jalview (58) and the amino acid sequences were colored based on charge (positive – blue, negative – red). Secondary structure predictions were made for each sequence using JPred online server (59) and all sequences were aligned based off of known annotated Loop 4 regions.

PyMOL (Version 3.1.3) was used for structural observations and alignments. Known structures of actin-bound, rigor Myo1B (PDB: 6C1H (39)), Myo1C (PDB: 9CFX (39)), and Myh11 (PDB: 6BIH (40)) along with actin-Tpm1.1 bound Myh7 (PDB: 6X5Z (41)) were aligned using PyMOL’s built-in align algorithm. The actin subunit on which the upper 50 KDa region of the myosin motor was bound was used as ground truth for alignment. AlphaFold3 was used to predict the structure of Myo19 using the full-length sequence from Uniprot (Q96H55) as well as full length Tpm1.7 and Tpm3.1. The first 450 amino acids of the predicted Myo19 structure were aligned to the first 450 amino acids of rigor Myo1B structure, these 450 amino acids roughly comprise the Upper 50 kDa portion of the myosin motors. The predicted structure of Tpm3.1 was aligned to the actin-bound Tpm1.1. The electrostatic surface of the alignef Tpm3.1 homodimer and Loop 4 of Myo19 and Myh11 were predicted using the PyMOL’s APBS electrostatics plugin (60).

### Graphing

Graphs and statistics were generated using GraphPad Prism 10.4.1 (GraphPad Software, Inc). Origin 2024b was used for the Mann–Whitney analysis of the GFP-Tpm3.1 fluorescence intensity decay.

## Data availability

Data for this study can be found at 10.5281/zenodo.18891749.

## Supporting information

This article contains supporting information.

## Conflict of interest

The authors declare that they have no conflicts of interest with the contents of this article.
