# Supplementary material for: Non-muscle tropomyosins inhibit myosin-19 and dynamically localize to mitochondrially associated actin filaments
Source: J Biol Chem. 2026 Mar 18;302(5):111377. doi: 10.1016/j.jbc.2026.111377 (PMC13092864; doi:10.1016/j.jbc.2026.111377)
Supplement: Supplementary Material 1 [file mmc1.pdf]

Supplementary Information

Non-muscle tropomyosins inhibit Myosin-19 and dynamically localize to mitochondrially-associated actin filaments

Cameron P. Thompson[1], Luther W. Pollard[1,2], Mengqi Xu[1],

Erika L.F. Holzbaur[1], E. Michael Ostap[1]

1. Department of Physiology and Pennsylvania Muscle Institute, Perelman School of Medicine, University of Pennsylvania, Philadelphia, Pennsylvania, USA
2. Biochemistry Department, University of Nebraska – Lincoln, Lincoln, Nebraska, USA

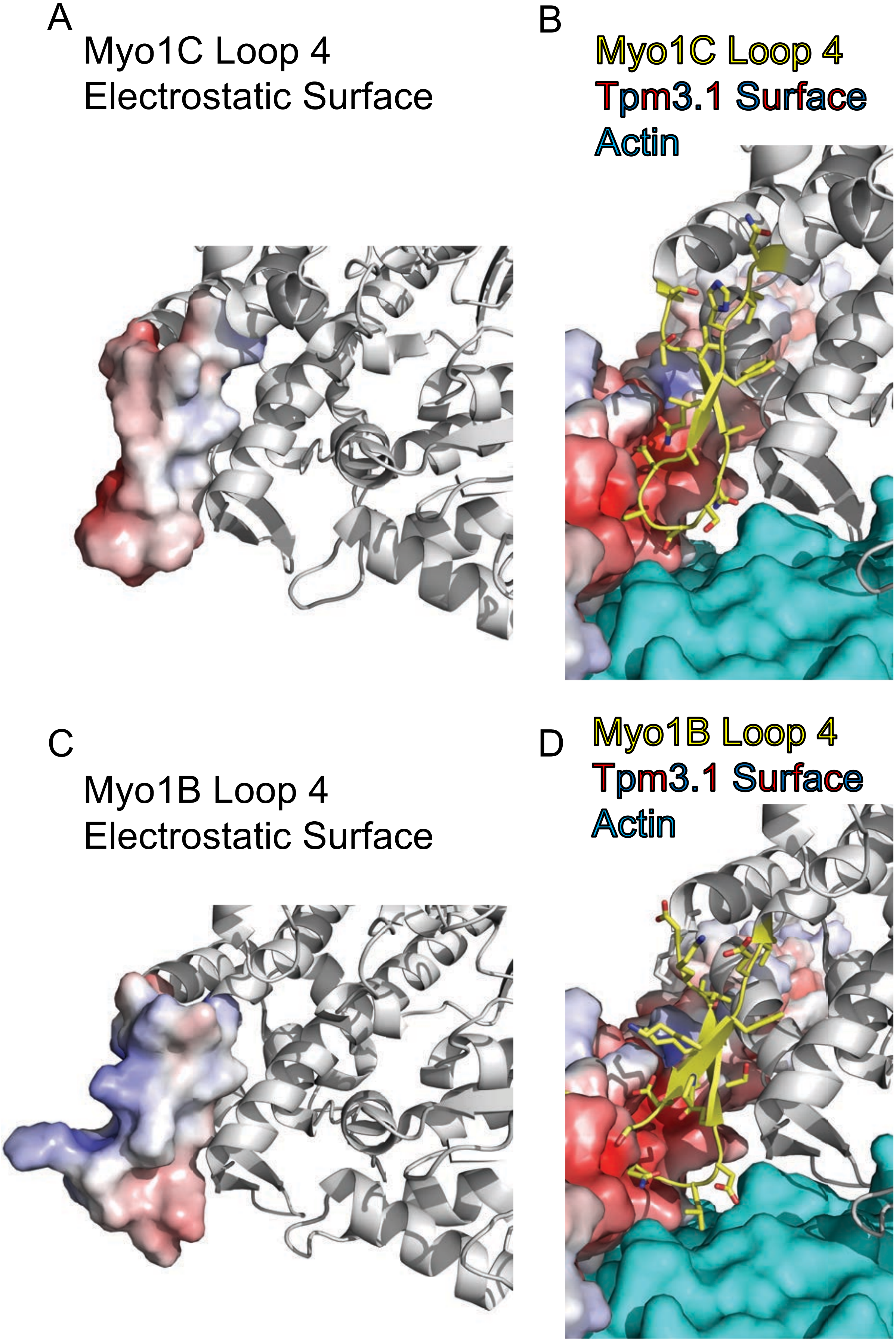

Supplementary Figure 1. A. Reported structure of rigor Myo1C Loop 4 (PDB: 9CFX[39]) and its modeled electrostatic surface. B. Aligned representation of reported Myo1C Loop 4 if bound to actin (cyan), and Tpm3.1 (electrostatic surface). C. Reported structure of rigor Myo1B Loop 4 (PDB: 6C1H[42]) as well as its modeled electrostatic surface. D. Aligned representation of reported Myo1B Loop 4 if bound to actin (cyan), and Tpm3.1 (electrostatic surface).

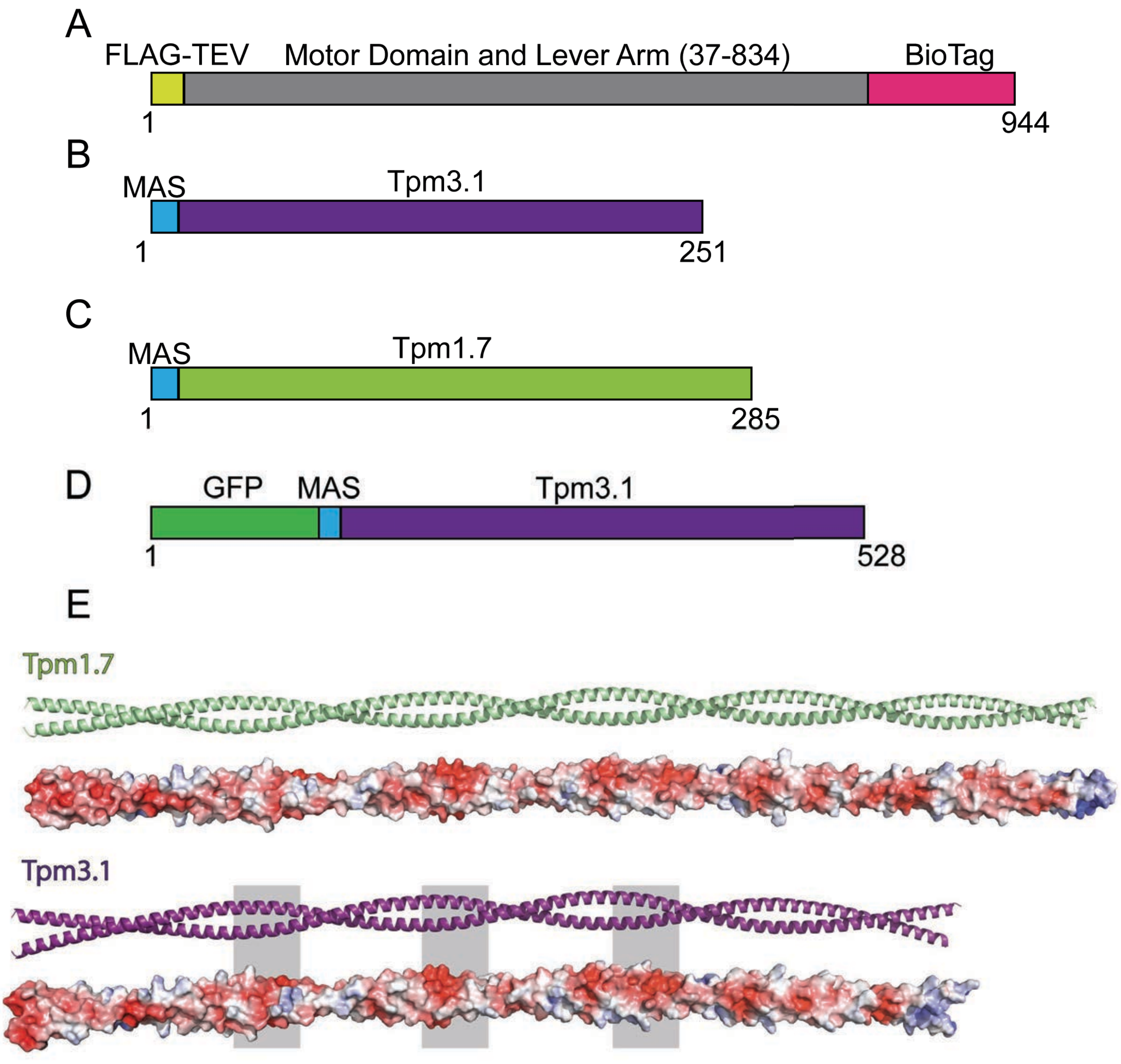

Supplementary Figure 2. Graphical Representation of Constructs used in Work. A. Myo19-3xIQ construct expressed and purified in SF9 cells, used for *in vitro* biochemistry experiments. B&C. Recombinant Tpm3.1 and Tpm1.7, containing N-terminal Met-Ala-Ser acetyl mimic sequence, expressed and purified in *E. coli*, used for *in vitro* biochemistry experiments. D. GFP linked to Tpm3.1 with Met-Ala-Ser linker either expressed and purified in *E. coli* for *in vitro* biochemistry experiments or transfected into HeLa cells for overexpression experiments. E. AlphaFold3 predicted structure of full-length Tpm1.7 (light green) and Tpm3.1 (purple) and their corresponding electrostatic surfaces. Grey, boxed regions on Tpm3.1 highlight predicted myosin interaction regions.

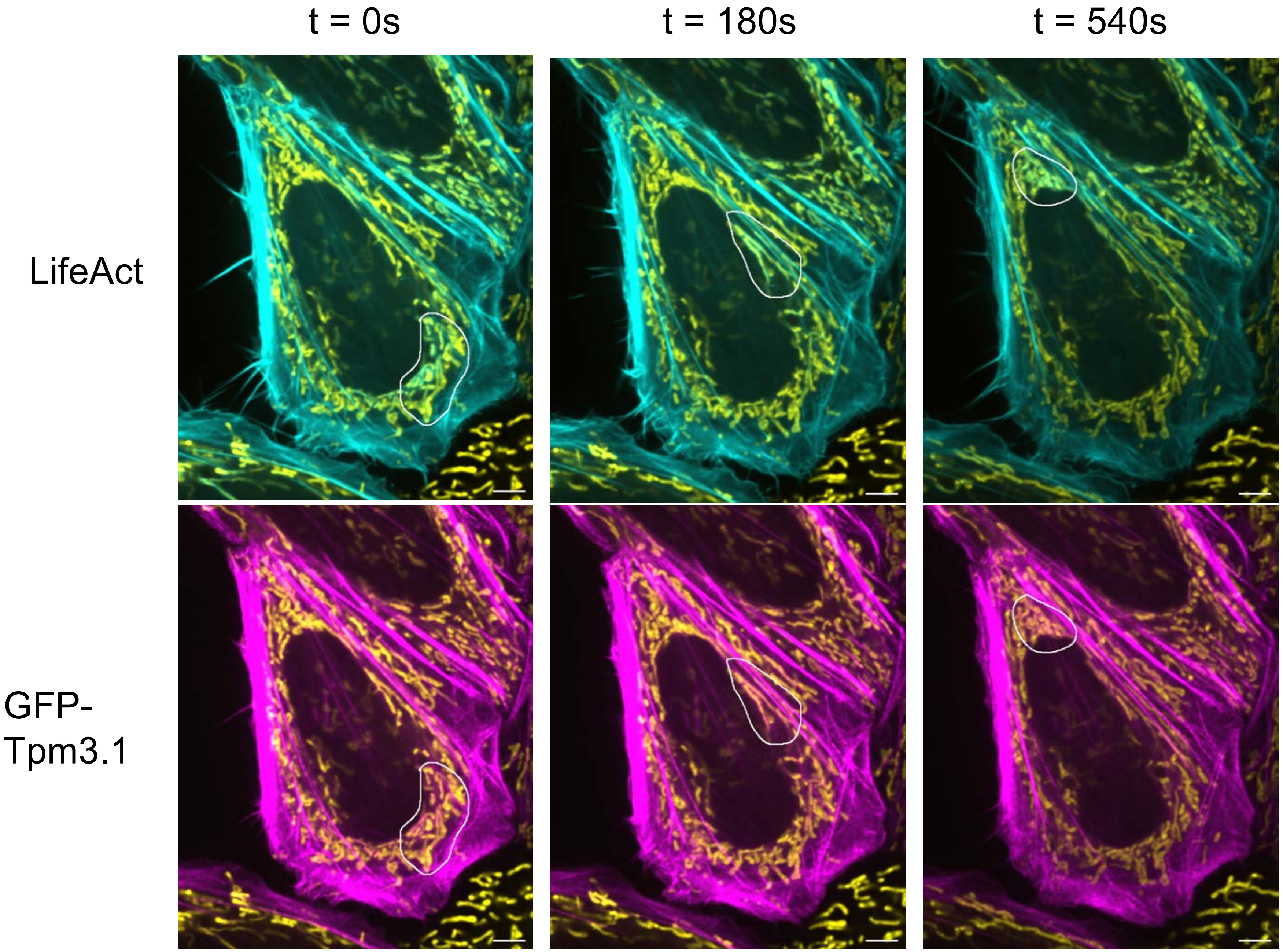

Supplementary Figure 3. Series of still images from the timelapse video in Video 4, circled region highlights the location of the actin wave (5 µm scale bar).

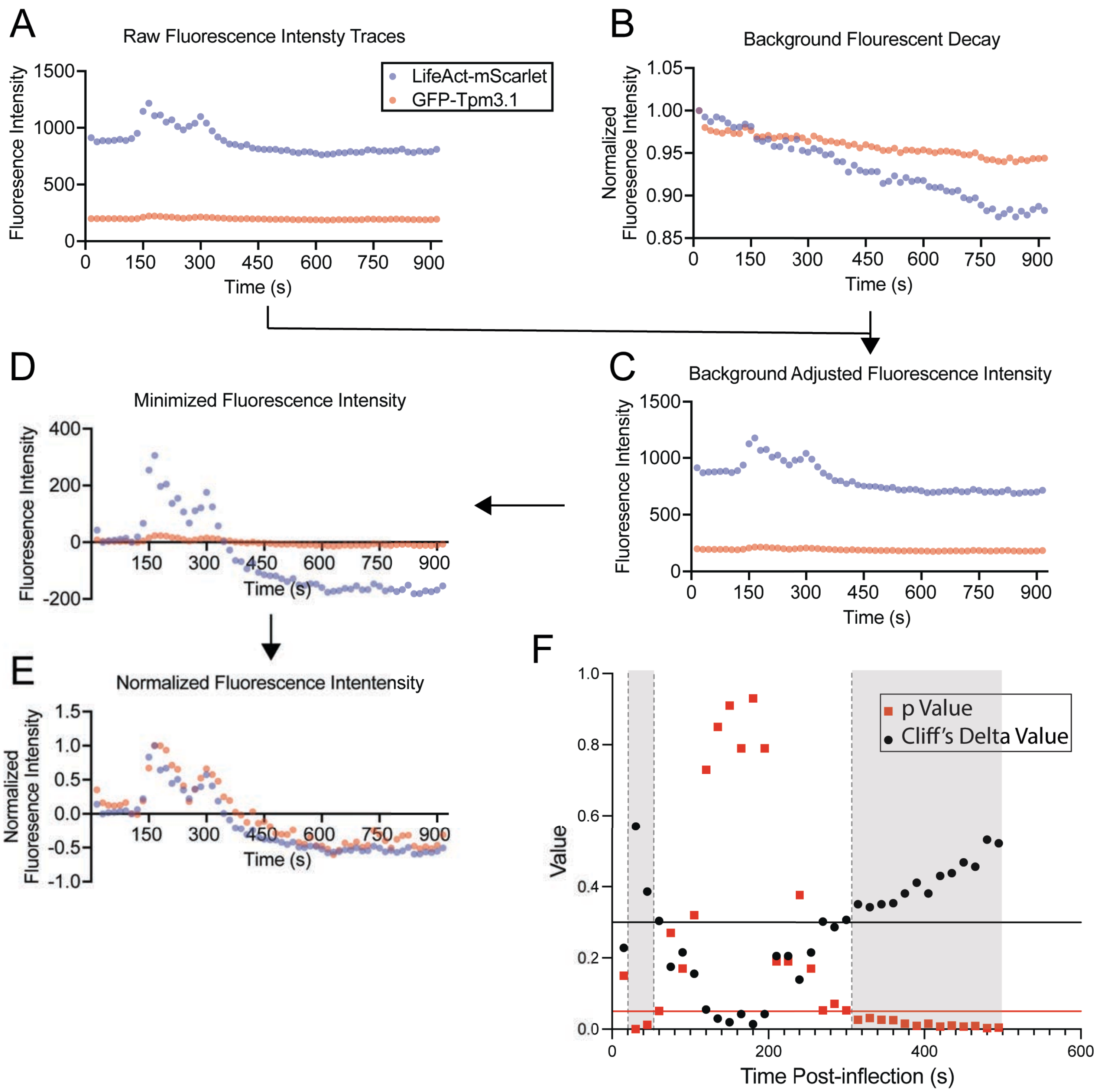

Supplementary Figure 4. Graphical representation of wave analysis correction, minimization, and normalization. A. Raw trace of LifeAct-mScarlett (blue) and GFP-Tpm3.1 (orange) fluorescence intensities. B. Full image background intensities of LifeAct and GFP-Tpm3.1 showing fluorescence decay throughout length of video. C. Trace resulting from wave signal correction by fraction of decay at each time point. D. Manual minimization to point prior to actin intensity increase. E. Normalization of both LifeAct and GFP-Tpm3.1 intensity traces to the maximum value for each. F. Graph of results from the Mann-Whitney test. Red squares represent the p-value of difference between the two curves at that timepoint, black dots represents the Cliff's Delta for the averages at the timepoint. Timepoints with values below the red line (0.05) and above the black line (0.3) are considered significantly different. The shaded grey regions denotes the timepoints where values are considered significantly different.

Video Figure Caption

Video 1. Actin gliding driven by surface bound Myo19-3xIQ. Captured at 37 ºC, 1 frame per second, 5 μm scale bar.

Video 2. Representative videos of various actin gliding assays performed across several concentrations of the same Myo19 preparation (1 frame per second, 5 μm scale bar). Videos were captured on the same experimental day from the same Myo19 preparation. Filament shredding is observed at high concentrations of motor, a common behavior when there are an abundance of surface bound motors.

Video 3. A. Time-lapse videos showing the movement of LifeAct (Left – Black and white, Right – Cyan) as the actin wave cycles through the cytoplasm and envelopes mitohcondria (Right – Yellow). B. Time-lapse videos showing the movement of GFP-Tpm3.1 (Left – Black and white, Right – Magenta) as the actin wave cycles through the cytoplasm and envelopes mitohcondria (Right – Yellow). 5 μm scale bar, 1 frame every 15 seconds.

Video 4. Time-lapse showing dynamic actin and Tpm3.1 wave traversing inset region in Figure 5A. Captured at 37 ºC, 4 frame per minute, 5 μm scale bar.
